# Integrating CTLA-4 Genetics and Soluble Isoforms for the Stratification of HCV-Related Hepatocellular Carcinoma Risk and Aggressiveness

**DOI:** 10.3390/ijms262211067

**Published:** 2025-11-15

**Authors:** Marwa Hassan, Walaa H. El-Maadawy, Sally A. Fahim, Sherihan M. Youssef, Omaima Mostafa Badran, Mahmoud Balata

**Affiliations:** 1 Immunology Department, Theodor Bilharz Research Institute, Giza 12411, Egypt; 2 Pharmacology Department, Theodor Bilharz Research Institute, Giza 12411, Egypt; w.elmadawy@tbri.gov.eg; 3 Biochemistry Department, School of Pharmacy, NewGiza University, Giza 12577, Egypt; 4 Pharmacy Practice Department, Faculty of Pharmacy, Delta University for Science and Technology, Gamasa 11152, Egypt; 5 Hepatogastoenterology Department, Theodor Bilharz Research Institute, Giza 12411, Egypt; 6 University Hospital Rostock, Ernst-Heydemann-Straße 6, 18057 Rostock, Germany; 7 University Hospital Bonn, Venusberg-Campus 1, 53127 Bonn, Germany

**Keywords:** biomarker, CTLA-4, gene polymorphism, hepatitis C virus, hepatocellular carcinoma, Immune checkpoint, rs231726, rs11571317, rs13384548

## Abstract

Host genetic factors influencing immune regulation are believed to modulate susceptibility to hepatitis C virus (HCV) and related hepatocellular carcinoma (HCC). This study aimed to investigate the association of Cytotoxic T-lymphocyte-associated antigen-4 (CTLA-4) genetic variants with HCV-related HCC risk, soluble CTLA-4 (sCTLA-4) levels, and disease severity. 225 age- and sex-matched participants (75 controls, 75 HCV, and 75 HCV-HCC) were enrolled. TaqMan allelic discrimination assays were used for genotyping three CTLA-4 SNPs, and sCTLA-4 was quantified by ELISA. Our results demonstrated that the rs231726 TT genotype and T-allele were significantly associated with HCC. The rs11571317 CC genotype and C-allele, alongside the rs13384548 GG genotype and G-allele, conferred increased risk for both HCV and HCC. Clinically, these high-risk genotypes correlated with worse liver function (Child–Pugh C), higher MELD/Na scores, and larger tumors. Moreover, sCTLA-4 levels showed a stepwise elevation from controls to HCV to HCC patients, peaking in carriers of the rs231726 TT and rs13384548 GG genotypes. In conclusion, this study identifies rs231726, rs11571317, and rs13384548 as robust genetic markers for HCV-related HCC susceptibility and cancer aggressiveness. Our findings provide novel evidence of their role in immune evasion through sCTLA-4 upregulation, offering new perspectives into genotype-based risk stratification and tailored immunotherapeutic strategies.

## 1. Introduction

Hepatocellular carcinoma (HCC) continues to pose a global health challenge, being the third leading cause of cancer-related mortality [1]. Chronic infection with the hepatitis C virus (HCV) is a major etiological driver, establishing a persistent inflammatory milieu that promotes progressive liver fibrosis, cirrhosis, and ultimately, hepatocarcinogenesis, especially in regions with high HCV prevalence like Egypt [2,3]. Despite the potential of direct-acting antiviral (DAA) therapies to reduce HCC incidence, a significant disease burden persists due to barriers to treatment and comorbidities such as alcohol use [4,5,6]. The substantial variability in outcomes among individuals indicates a critical, unresolved role for host genetic determinants in shaping disease susceptibility and progression [7,8].

Among these determinants, immune checkpoint pathways that regulate T-cell activation have emerged as crucial players in both antiviral defense and cancer immunosurveillance [9,10,11]. Cytotoxic T-lymphocyte-associated antigen-4 (CTLA-4), expressed on activated T cells, is an inhibitory receptor that dampens the T-cell reactions by competing with the co-stimulatory receptor CD28 for its ligands, thereby acting as an essential brake on the immune response [12,13]. This mechanism is not only exploited by malignancies to evade immune eradication, but it also serves as a proven therapeutic strategy, with CTLA-4 blockade demonstrating effectiveness in oncology [14]. Beyond its membrane-bound counterpart, a soluble isoform of CTLA-4 (sCTLA-4), generated by alternative splicing, retains immunomodulatory capacity. It has been implicated in the suppression of T-cell activation in autoimmunity and cancer, suggesting its potential as a biomarker for immune system dysfunction [15].

Genetic variations within the CTLA-4 gene, such as rs231726, rs11571317 (a promoter variant), and rs13384548 (positioned in the 3′-UTR with putative miRNA regulatory role), can profoundly affect its expression and function. They have been mechanistically linked to altered CTLA-4 activity in immunological disorders [16,17,18]. Previous investigations on CTLA-4 genetics in HCC have yielded incongruous results, focusing on a limited array of SNPs without exploring their functional consequences on soluble protein levels. The possible impact of these polymorphisms on the incidence of HCV persistence and the subsequent transition to HCC within high-risk populations, such as those in Egypt, remains poorly understood. This gap is significant, as a genetic predisposition towards immune suppression could promote viral persistence and create a conducive milieu for tumorigenesis [19].

Therefore, this study was designed to address these critical gaps and provide novel insights. To our knowledge, this is the first comprehensive study to simultaneously investigate the association of the three functional SNPs, rs231726, rs11571317, and rs13384548, with the risk of HCV-related HCC, while concurrently integrating these genetic findings with circulating sCTLA-4 levels and clinicopathological features. Our work aimed to identify novel molecular biomarkers that could refine risk and disease aggressiveness stratification and open avenues for personalized immunotherapeutic interventions in HCC.

## 2. Results

### 2.1. Genes Correlated with HCC

We identified 10,000 genes associated with HCC and selected the top 170 based on the highest GIFtS and relevance scores. Among these, CTLA-4 was included, highlighting its strong association with HCC. Proteins with high GIFtS values are considered biologically significant, as they indicate extensive functional annotation and substantial research evidence linking them to essential cellular mechanisms or disease pathways.

### 2.2. Protein–Protein Interactions

**Figure 1:**
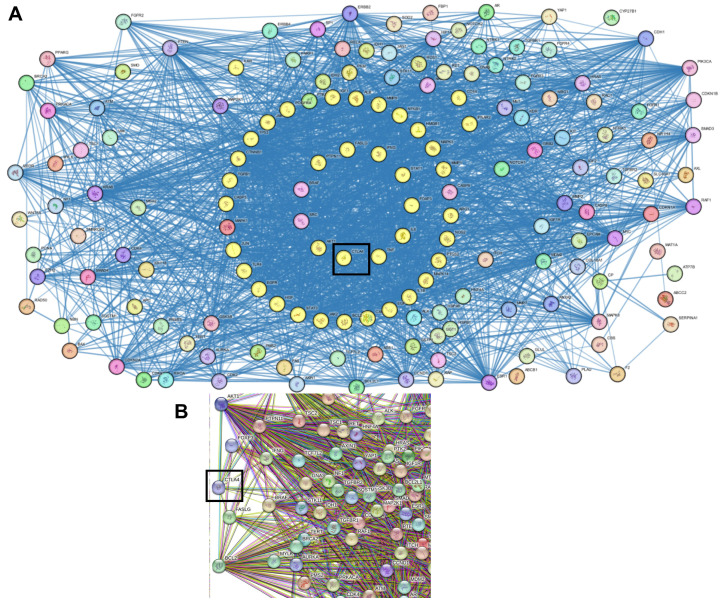
STRING Protein–protein interaction analyses. The middle yellow circles represent CTLA-4 connected to HCC-related proteins. The thickness of the connecting edges denotes the strength of interaction (**A**), while the colored edges represent the type of interaction (**B**).

At a confidence threshold of 0.7, STRING PPI analysis of the top 170 genes generated a densely connected network (clustering coefficient: 0.56) comprising 167 nodes and 2069 edges, markedly higher than the expected 530 edges. This reflects a significantly enriched level of protein–protein interactions compared to random gene sets (*p* < 0.0001). In the network, edge thickness denotes interaction confidence (Figure 1A), while edge colors represent the type of interaction (Figure 1B). Notably, CTLA-4 shows multiple connections with HCC-related proteins, supported by diverse evidence sources such as co-expression, literature mining, experimental validation, and curated databases.

### 2.3. Demographic and Clinical Data of the Study Subjects

The three examined groups showed no significant differences regarding age (*p* = 0.500), gender distribution (*p* = 0.688), smoking status (*p* = 0.396), or the prevalence of diabetes mellitus (*p* = 0.618). However, marked biochemical disparities were seen in laboratory investigations, with the HCV-HCC cohort demonstrating significantly elevated liver injury markers, including AST, ALT, and total bilirubin, in comparison to both controls and HCV patients (*p* < 0.001). This group also had substantially compromised liver function, as evidenced by a considerable decline in serum albumin and prothrombin levels with disease development. Notably, AFP levels demonstrated a dramatic rise in HCV-HCC patients compared to controls and HCV-only patients (*p* < 0.001), underscoring its robust association with HCC development (Table 1).

### 2.4. Genotyping of CTLA-4 Variants

Analysis of genotype and allele distributions revealed that for rs231726, the frequencies of genotypes varied significantly throughout the studied groups (*p* < 0.001). The distribution in HCV patients was comparable. In contrast, HCC patients exhibited a marked shift toward the TT genotype, detected in 57.3% of cases, with CC and CT observed in 18.7% and 24.0%, respectively (*p* < 0.001). The TT genotype alone was significantly correlated with HCC susceptibility, yielding an OR of 7.06 (95% CI: 3.27–15.21, *p* < 0.001) and 5.86 (95% CI: 2.80–12.26, *p* < 0.001) when compared to the control and HCV groups, respectively. However, the CC genotype seemed to have a protective effect. At the allele level, the T-allele frequency increased from 34.0% in controls and 36.3% in HCV to 69.3% in HCC patients (OR = 4.389, 95% CI: 2.70–7.12, *p* < 0.001; OR = 3.795, 95% CI: 2.35–6.13, *p* < 0.001, respectively) (Table 2).

The rs11571317 polymorphism displayed a divergent genotype distribution amongst the groups. The controls had a high frequency of the TT genotype (68.00%), whereas patients with HCV and HCC showed a significant reduction (10.70% and 21.33%, respectively, *p* < 0.001). Conversely, the CC genotype was rare in controls (6.70%) but overrepresented in HCV and HCC patients (49.30% and 41.33%, respectively; *p* < 0.001). It was strongly associated with risks of HCV infection and HCC development (OR = 13.63, 95% CI: 4.95–37.58 and OR = 9.86, 95% CI: 3.57–27.27, respectively), while the TT genotype appeared protective. Allelic analysis demonstrated a higher prevalence of the C-allele in HCV (69.30%) and HCC (60.00%) than in controls (19.30%) (*p* < 0.001), further supporting its role in disease predisposition (Table 3).

The rs13384548 polymorphism also showed notable differences in genotype distribution across groups (*p* < 0.001). The frequency of the GG genotype increased progressively in HCV (45.3%) and reached its peak in HCC patients (69.3%), while the AA genotype decreased sharply to 9.3% in cases of HCC. Individuals carrying the GG genotype had an elevated HCV risk by 3.61-fold (95% CI: 1.73–7.56, *p* < 0.001) and HCC risk by 9.85-fold (95% CI: 4.61–21.07, *p* < 0.001). Furthermore, HCV patients having the GG genotype are more prone to develop HCC (OR = 2.73, 95% CI: 1.40–5.32, *p* = 0.003). On the other hand, the GA and AA genotypes were less frequent in HCC than in controls and conferred a protective effect. The G-allele was significantly enriched in HCV (60.7%) and HCC (80.0%) versus controls (40.0%) (*p* < 0.001), reinforcing its correlation with heightened HCV risk (OR = 2.31, 95% CI: 1.46–3.68, *p* < 0.001), relative to controls and increased HCC susceptibility (OR = 6.00, 95% CI: 3.58−10.06, *p* < 0.001 and OR = 2.59, 95% CI: 1.55–4.35, *p* < 0.001) in comparison to the healthy subjects and HCV patients, respectively (Table 4).

Analysis of the HCC clinicopathological parameters in relation to genotypes indicated that certain variants were substantially correlated with disease severity. For rs231726, the TT genotype was significantly associated with poorer liver function, as evidenced by the highest proportion of Child–Pugh class C (55.8%, *p* = 0.006), intermediate MELD/Na scores (*p* = 0.023), and increased tumor size (*p* < 0.001). Similarly, the rs11571317 CC genotype correlated with elevated MELD/Na scores (*p* = 0.006) and larger lesions (*p* = 0.002), but not with Child–Pugh or BCLC stage. The rs13384548 GG genotype was linked to worse Child–Pugh scores (50.3% class C), MELD/Na stratification (*p* = 0.001), BCLC staging (*p* = 0.028), and tumor size (*p* = 0.031). However, none of the studied SNPs exhibited a significant relationship with tumor multiplicity (Table 5).

### 2.5. Measurements of Soluble CTLA-4 (sCTLA-4)

**Figure 2:**
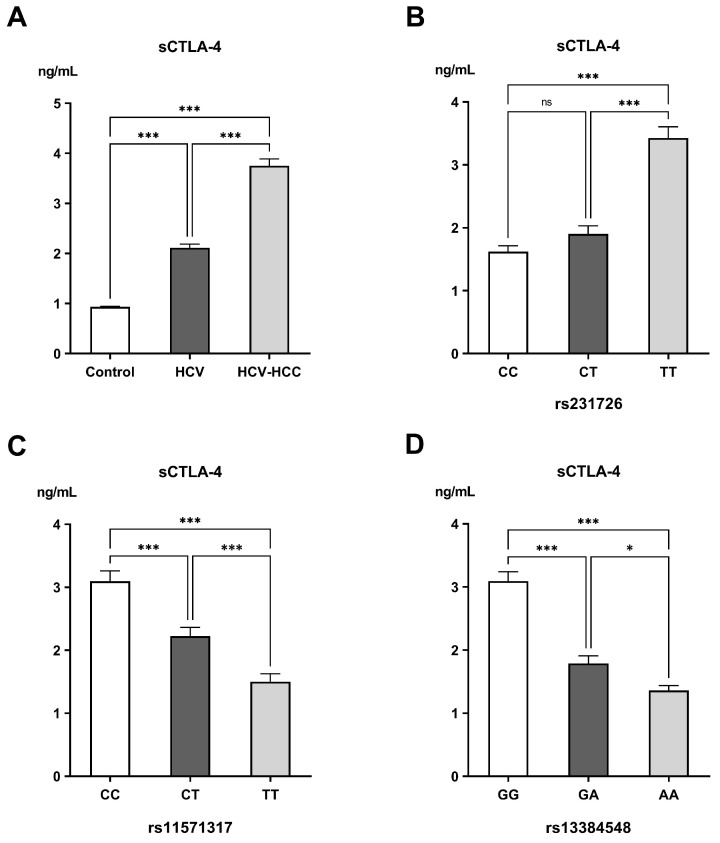
Levels of soluble CTLA-4 (sCTLA-4) in the different (**A**) examined groups, (**B**) genotypes of rs231726, (**C**) genotypes of rs11571317, and (**D**) genotypes of rs13384548. ns: non-significant difference; * Significant difference with *p* < 0.05; *** significant difference with *p* < 0.001.

Serum concentrations of sCTLA-4 demonstrated a progressive and highly significant elevation from the control group (0.93 ± 0.011 ng/mL) to the HCV group (2.12 ± 0.07 ng/mL), reaching the highest levels in the HCV-HCC group (3.75 ± 0.14 ng/mL; *p* < 0.001). Genotypic analysis revealed noticeable differences in sCTLA-4 expression. For rs231726, sCTLA-4 levels dramatically rose from 1.62 ± 0.09 ng/mL in CC carriers to 1.90 ± 0.13 ng/mL in CT and 3.43 ± 0.18 ng/mL in TT carriers (*p* < 0.001). For rs11571317, individuals harboring the CC genotype had the greatest sCTLA-4 concentration (3.10 ± 0.16 ng/mL), followed by those having the CT (2.23 ± 0.14 ng/mL) and TT genotypes (1.50 ± 0.13 ng/mL, *p* < 0.001). Carriers of the GG genotype for rs13384548 had markedly elevated sCTLA-4 levels (3.09 ± 0.15 ng/mL) compared with those with the GA (1.79 ± 0.12 ng/mL) and AA genotypes (1.36 ± 0.07 ng/mL, *p* < 0.001) (Figure 2). When correlated with clinical parameters, higher sCTLA-4 concentrations were observed in patients with compromised liver function (Child–Pugh class C). A strong, non-significant trend was also noted towards increased sCTLA-4 levels in patients with larger tumors, whereas no significant associations were found with MELD/Na score, BCLC stage, or frequency of tumor lesions (Table 5).

**Table 5:** Relationship of genetic polymorphisms and soluble CTLA-4 (sCTLA-4) with clinicopathological features in hepatocellular carcinoma (HCC) patients.

	rs231726 TT n = 43	*p*-Value	rs11571317 CC n = 31	*p*-Value	rs13384548 GG n = 52	*p*-Value	sCTLA-4	*p*-Value
**Child–Pugh score**								
A	8 (18.60%)	0.006	5 (16.10%)	0.086	11 (21.20%)	0.031	3.37 ± 0.29	0.031
B	11 (25.60%)		11 (35.50%)		15 (28.80%)		3.48 ± 0.22	
C	24 (55.80%)		15 (48.40%)		26 (50.30%)		4.15 ± 0.20	
**MELD/Na score**								
≤9	4 (9.30%)	0.023	2 (6.50%)	0.006	4 (7.70%)	0.001	3.41 ± 0.32	0.479
10–19	17 (39.50%)		14 (45.20%)		21 (40.40%)		4.02 ± 0.24	
20–29	14 (32.60%)		11 (35.50%)		19 (36.50%)		3.66 ± 0.23	
≥30	8 (18.60%)		4 (12.90%)		8 (15.40%)		3.72 ± 0.40	
**BCLC stage**								
A	5 (11.60%)	0.209	3 (9.70%)	0.110	6 (11.50%)	0.028	3.11 ± 0.39	0.226
B	14 (32.60%)		11 (35.50%)		21 (40.40%)		3.95 ± 0.24	
C	13 (30.20%)		11(35.50%)		14 (26.90%)		3.76 ± 0.23	
D	11 (25.60%)		6 (19.40%)		11 (21.20%)		3.91 ± 0.31	
**Number of lesions**								
<3	21 (48.80%)	0.879	15 (48.40%)	0.857	28 (53.80%)	0.579	3.69 ± 0.21	0.661
≥3	22 (51.20%)		16 (51.6%)		24 (46.20%)		3.82 ± 0.17	
**Largest lesion**								
<3 cm	10 (23.30%)	<0.001	7 (22.6%)	0.002	17 (32.70%)	0.013	3.40 ± 023	0.073
≥3 cm	33 (76.70%)		24 (77.4%)		35 (67.30%)		3.92 ± 0.16	

Quantitative data are listed as frequencies (percentages). Qualitative data are listed as mean ± standard error (SE).

## 3. Discussion

This study provides compelling evidence that inherited variations in the CTLA-4 immune checkpoint gene establish a genetic predisposition for both HCV persistence and subsequent HCC by promoting a state of progressive immune dysregulation. This is demonstrated by the finding that high-risk genotypes for three functional SNPs, rs231726 TT, rs11571317 CC, and rs13384548 GG, are linked to elevated levels of sCTLA-4 and more aggressive clinicopathological features of HCC, suggesting their potential utility as biomarkers for risk stratification and prognosis.

HCC represents one of the leading causes of cancer-related mortality globally, with chronic HCV infection serving as a major predisposing factor [20,21]. While viral, environmental, and metabolic factors are involved in the process of hepatocarcinogenesis, the influence of host genetic determinants on shaping immune responses and susceptibility to HCC remains less well explored [22,23]. Among these, immune checkpoint molecules such as CTLA-4 have garnered considerable attention for their pivotal role in T-cell regulation and their therapeutic relevance as targets for immune checkpoint inhibitors [24].

The Ras association domain family 1 isoform A (RASSF1A), CTLA-4, and signal transducer and activator of transcription 4 (STAT4) genes are involved in the regulation of the cell cycle, apoptosis, and the autoimmune response against cancer. The activation of the immune checkpoint gene CTLA4 or its polymorphisms causes phosphorylation of kinases essential for RAS gene activation. This, in turn, downregulates the tumor suppressor RASSF1, inhibiting apoptosis and leading to HCC development, indicating a detrimental impact of CTLA4 gene polymorphism on HCV-associated HCC cases [25].

Although CTLA-4 gene polymorphisms and sCTLA-4 have been investigated in autoimmune diseases and a few malignancies, their contribution to HCC development in the context of HCV infection remains poorly defined. To our knowledge, this is among the first studies to thoroughly examine the association between CTLA-4 gene polymorphisms and the risk and progression of HCV and HCC, in conjunction with circulating sCTLA-4 levels in the Egyptian population.

The rs231726 lies in the vicinity of the CTLA-4/ICOS locus on chromosome 2q33, a few kilobases downstream of its 3′-UTR. Our study demonstrated a strong relationship between the rs231726 TT genotype and the T-allele and increased HCC risk and tumor size. This result agrees with previous research indicating that the rs231726 is linked to systemic lupus erythematosus and alopecia areata [18,26]. These findings suggest that the T-allele may enhance CTLA-4 expression, resulting in increased inhibitory signaling and diminished T-cell activation, a state that favors tumor immune escape. Our observation that carriers of this genotype also had higher sCTLA-4 levels substantiates this hypothesis, indicating that genetic variation may influence not only membrane-bound CTLA-4 expression but also its soluble isoform, which has been implicated in immunological dysregulation in malignancies.

In the present study, we observed that the CC genotype and C-allele of the rs11571317 (−658 C/T) variant were associated with both HCV persistence and subsequent HCC development, while the TT genotype and T-allele appeared protective. Previous research on this polymorphism is scarce; however, similar trends have been reported for CTLA-4 promoter variants, which are known to affect the expression levels of alternatively spliced full-length CTLA-4 and sCTLA-4 isoforms [27,28]. Also, the rs11571317 T-allele has been linked to reduced transcriptional activity due to abolition of the SP-1 binding site, and consequently lower inhibitory signaling [29]. The correlation of the CC genotype with chronic HCV infection and hepatocarcinogenesis in our study aligns with prior studies, where increased CTLA-4 expression is associated with impaired immune responses to breast cancer [17,29]. Thus, our findings suggest that rs11571317 may modulate host susceptibility to both viral persistence and malignant transformation by influencing CTLA-4 transcriptional regulation.

The rs13384548 is situated in the 3′-UTR of the CTLA-4 gene, positioning it to presumably influence the post-transcriptional regulation of CTLA-4 mRNA. In addition, a pivotal functional study revealed that it interferes with the miR-302a binding site within the CTLA-4 3′-UTR, reducing the capacity of miR-302a to regulate CTLA-4 mRNA stability or translation, potentially leading to elevated CTLA-4 expression [16]. Although prior publications don’t explicitly associate rs13384548 with clinical traits, its biological function in miRNA-mediated regulation offers a plausible mechanism for affecting illness risk and progression. Our study is the first to demonstrate a clear clinical association linking rs13384548 to both HCV susceptibility and HCC severity. Specifically, individuals who possess the GG genotype had heightened risk and features of more aggressive hepatocarcinogenesis, including worse liver function, more advanced Child–Pugh score and BCLC stage, as well as larger tumors. This underscores the possible involvement of this polymorphism in the pathophysiology of HCV-related HCC and disease aggressiveness. The compelling association of the rs13384548 GG genotype with HCV-HCC risk and severity stands in contrast to our previous findings in pancreatic ductal adenocarcinoma (PDAC), where the same polymorphism did not demonstrate a significant association with disease risk [30]. This marked discrepancy underscores that the role of CTLA-4 polymorphisms is not uniform across all cancer types but is likely highly context-dependent, influenced by the distinct immunobiology of the underlying illness. The chronic inflammatory and virally driven microenvironment of HCV-related hepatocarcinogenesis may create a distinct selective pressure wherein CTLA-4-mediated immune dysregulation becomes a critical driver of disease progression, a mechanism that appears less pivotal in the pathogenesis of PDAC.

One of the most striking findings of our study is the continuing elevation of circulating sCTLA-4 levels in HCV and HCC patients, particularly among those classified as advanced Child–Pugh class. This establishes sCTLA-4 as a biomarker that escalates with disease progression from health to chronic infection to overt malignancy. Elevated sCTLA-4 has been previously documented in chronic hepatitis, cirrhosis, autoimmunity, and different solid tumors, where it correlates with immune suppression and poor prognosis [15,31,32,33]. In line with these reports, we found that sCTLA-4 levels were not only higher in HCC compared to controls but also linked to certain CTLA-4 genotypes, indicating a genetic basis for interindividual variability in sCTLA-4 production and highlighting the intricacy of genotype-phenotype interactions in HCC. Functionally, sCTLA-4 facilitates immune evasion by competitively inhibiting CD28-mediated T-cell activation, thus dampening antitumor immunity [24]. This confirms the potential of sCTLA-4 as both a mediator of immune suppression in HCC and a biomarker of disease severity.

Our results, which link specific high-risk CTLA-4 genotypes to elevated sCTLA-4 levels and disease severity, provide a plausible genetic explanation for the observed efficacy of CTLA-4 blockade in a subset of HCC patients. This is illustrated by the clinical activity of the anti-CTLA-4 monoclonal antibody Tremelimumab. A phase I/II study including patients with advanced HCV-related HCC has demonstrated that Tremelimumab not only exhibits a controllable safety profile and antitumor activity but also displays significant antiviral effects, reducing viral load in a number of patients [34]. This suggests that patients harboring the high-risk genotypes identified in our study, who have upregulated CTLA-4 pathway activity, might be the ideal candidates to gain the most benefit from such immunotherapeutic interventions.

Despite the significant associations identified, the current study is not without limitations. The sample size, although sufficient to detect significant associations, remains relatively modest, necessitating replication in larger multicenter cohorts. Moreover, the single-center, ethnically specific (Egyptian) nature of our cohort, while a strength for studying a high-risk population, may limit the generalizability of our results to other ethnic groups. Future longitudinal studies, complemented by functional experiments, are essential to confirm the prognostic value of these biomarkers and elucidate the underlying molecular mechanisms, particularly in the era of immune checkpoint inhibitors and direct-acting antivirals.

The findings of this study carry significant clinical implications for the management of HCV-related HCC. The identification of high-risk genotypes (rs231726 TT, rs11571317 CC, and rs13384548 GG) could serve as a valuable tool for improving risk stratification, enabling physicians to prioritize intensified surveillance for genetically susceptible individuals and HCV patients. Furthermore, the association between rs231726 TT, rs11571317 CC, and rs13384548 GG and elevated sCTLA-4 suggests that these patients may have enhanced responsiveness to CTLA-4 blockade and combination treatments that counteract suppression. These genotype-specific insights pave the way for personalized HCC prevention and treatment strategies, potentially optimizing outcomes through tailored therapeutic approaches. Future studies should explore whether genetic testing for these variants, in conjunction with sCTLA-4 monitoring, can guide clinical decision-making in real-world settings.

## 4. Materials and Methods

### 4.1. Study Population and Design

This case–control study comprised three age- and gender-matched cohorts: 75 healthy adults (control group), 75 patients with chronic HCV infection, and 75 patients with HCV-related HCC. All patients were sourced from the Hepato-Gastroenterology Clinic and Department at Theodor Bilharz Research Institute. The diagnosis of chronic HCV infection was based on seropositivity for anti-HCV antibodies and the presence of detectable HCV RNA by PCR for at least six months. The diagnosis of HCC was established via distinctive imaging findings on triphasic CT and/or MRI. Exclusion criteria encompassed co-infection with the hepatitis B virus (HBV) or HIV, other etiologies of chronic liver disease (such as alcoholic liver disease and Wilson’s disease), metastatic liver tumors, and a prior history of chemotherapy or transplantation.

Demographic information, including age, gender, smoking status, and prevalence of diabetes mellitus, was recorded for all participants. Also, the subjects underwent laboratory assessments, which involved liver and renal function tests and fasting blood glucose measurements. AFP levels were quantified by enzyme-linked immunosorbent assay (ELISA). The severity of disease in HCC patients was evaluated utilizing the Child–Pugh classification, the Model for End-Stage Liver Disease Sodium score (MELD/Na), and the Barcelona Clinic Liver Cancer (BCLC) staging system. Tumor burden was determined according to the number (<3 vs. ≥3) and size of focal lesions (<3 cm vs. ≥3 cm) using abdominal imaging.

### 4.2. Retrieving Genes Correlated with HCC

HCC-associated genes were retrieved from the GeneCards database [35]. The top genes with the highest GeneCards Inferred Functionality Scores (GIFtS) and relevance scores were selected, as these metrics indicate strong functional importance and disease relevance, suggesting a pivotal role in HCC pathogenesis and potential therapeutic value.

### 4.3. Gene Ontology

GO and KEGG enrichment analyses were performed to explore the biological functions of the selected genes, with ShinyGO used to generate lollipop plots of enriched processes and pathways [36].

### 4.4. Genotyping of CTLA-4 Variants

Genomic DNA was extracted from blood samples using a commercial DNA extraction kit (Thermo Fisher Scientific, Waltham, MA, USA) following the manufacturer’s protocol. The concentration and purity of DNA were assessed using the NanoDrop spectrophotometer (Thermo Fisher Scientific, Waltham, MA, USA). Genotyping of the rs231726 (C > T), rs11571317 (C > T), and rs13384548 (G > A) polymorphisms was performed using TaqMan allelic discrimination real-time PCR assays (Applied Biosystems, Foster City, CA, USA). PCR amplification was carried out using TaqMan™ Genotyping Master Mix (Thermo Fisher Scientific, Waltham, MA, USA) under the following cycling parameters: initial enzyme activation at 95 °C for 10 min, and 40 cycles of denaturation at 95 °C for 15 s and annealing/extension at 60 °C for 1 min. Allelic discrimination was conducted automatically using the StepOnePlus Real-Time PCR System (Thermo Fisher Scientific, Waltham, MA, USA).

### 4.5. Measurement of Soluble CTLA-4 (sCTLA-4)

Serum concentrations of soluble cytotoxic T-lymphocyte-associated antigen-4 (sCTLA-4) were quantified with a commercially available human sCTLA-4 ELISA kit (Thermo Fisher Scientific, Waltham, MA, USA) according to the manufacturer’s guidelines.

### 4.6. Statistical Analysis

Data were analyzed utilizing SPSS version 25 (SPSS Inc., Chicago, IL, USA). Continuous variables were expressed as mean ± standard error (SE) and compared using one-way ANOVA followed by post hoc LSD tests. Categorical variables were represented as frequencies and percentages and analyzed using the Chi-square test, with the Bonferroni correction applied for multiple comparisons. Hardy–Weinberg equilibrium (HWE) was tested in the control group for each SNP. Genetic associations were evaluated by calculating odds ratios (ORs) with 95% confidence intervals (CIs). A post hoc power analysis was performed to verify the achieved power for the genetic associations identified in this study. The analysis demonstrated over 80% power to identify relationships at a significant level of 0.05 for the analyzed SNPs. Associations between SNPs and clinicopathological features were assessed using the Chi-square test. A two-tailed *p*-value of less than 0.05 defined statistical significance.

## Figures and Tables

**Table 1:** Demographic and laboratory characteristics of the study participants.

		Groups		*p*-Value
	Control (n = 75)	HCV (n = 75)	HCV-HCC (n = 75)	
**Age (year)**	59.93 ± 0.93	61.36 ± 0.82	60.67 ± 0.802	0.500
**Gender**				
Males	36 (48.00%)	35 (46.67%)	40 (53.33%)	0.688
Females	39 (52.00%)	40 (53.33%)	35 (46.67%)	
**Smoking**				
No	40 (53.33%)	42 (56%)	34 (45.33%)	0.396
Yes	35 (46.67%)	33 (44%)	41 (54.67%)	
**Laboratory data**				
AST (IU/L)	23.43 ± 0.874	54.87 ± 3.719	195.17 ± 33.326 ^#^	<0.001
ALT (IU/L)	24.68 ± 1.517	47.37 ± 2.78 **	86.63 ± 9.97 ^#^	<0.001
Total bilirubin (mg/dL)	0.59 ± 0.03	2.55 ± 0.43 *	6.45 ± 1.00 ^#^	<0.001
Serum albumin (g/dL)	4.07 ± 0.05	3.40 ± 0.09 ***	2.48 ± 0.06 ^#^	<0.001
PC (%)	95.86 ± 0.78	76.18 ± 2.29 ***	57.65 ± 2.41 ^#^	<0.001
AFP (ng/mL)	4.42 ± 0.21	8.57 ± 0.53	16,051.87 ± 3463.00 ^#^	<0.001

Qualitative data are expressed as frequencies (percentages). Quantitative data are expressed as mean ± standard error (SE). * Significant change compared to the control group (*p* < 0.05). ** Significant change compared to the control group (*p* < 0.01). *** Significant change compared to the control group (*p* < 0.001). ^#^ significant change compared to the control and HCV groups (*p* < 0.001). HCC: Hepatocellular carcinoma; AST: Aspartate aminotransferase; ALT: Alanine aminotransferase; PC: Prothrombin concentration; AFP: Alpha-fetoprotein.

**Table 2:** Genotype and allele frequencies of rs231726 (C > T) SNP and association with risk of hepatocellular carcinoma (HCC) development.

	Control (n = 75)	HCV (n = 75)	HCV-HCC (n = 75)	*p*-Value	Odds Ratio (95% CI) of HCC vs. Control	*p*-Value	Odds Ratio (95% CI) of HCC vs. HCV	*p*-Value
**Genotype**								
CC	36 (48.00%)	33 (44.00%)	14 (18.70%) ^#^	<0.001				
CT	27 (36.00%)	28 (37.30%)	18 (24.00%)					
TT	12 (16.00%)	14 (18.70%)	43 (57.30%) ^#^					
**Genotype**								
CC	36 (48.00%)	33 (44.00%)	14 (18.70%) ^#^	<0.001	0.25	<0.001	0.29	0.001
CT + TT	39 (52.00%)	42 (56.00%)	61 (81.30%) ^#^		(0.12–0.52)		(0.14–0.61)	
**Genotype**								
CT	27 (36.00%)	28 (37.30%)	18 (24.00%)	0.158	0.56	0.109	0.53	0.077
CC + TT	48 (64.00%)	47 (62.70%)	57 (76.00%)		(0.28–1.14)		(0.26–1.08)	
**Genotype**								
TT	12 (16.00%)	14 (18.70%)	43 (57.30%) ^#^	<0.001	7.06	<0.001	5.86	<0.001
CC + CT	63 (84.00%)	61 (81.30%)	32 (42.70%) ^#^		(3.27–15.21)		(2.80–12.26)	
**Allele**								
C	99 (66.00%)	94 (62.70%)	46 (30.70%) ^#^	<0.001	0.23	<0.001	0.26	<0.001
T	51 (34.00%)	56 (36.30%)	104 (69.30%) ^#^		(0.14–0.37)		(0.16–0.43)	

The data are listed as frequencies (percentages). ^#^ Significant change compared to the control and HCV groups (*p* < 0.001).

**Table 3:** Genotype and allele frequencies of rs11571317 (C > T) SNP and association with risks of hepatitis C virus (HCV) and hepatocellular carcinoma (HCC) development.

	Control (n = 75)	HCV (n = 75)	HCV-HCC (n = 75)	*p*-Value	Odds Ratio (95% CI) of HCV vs. Control	*p*-Value	Odds Ratio (95% CI) of HCC vs. Control	*p*-Value
**Genotype**								
CC	5 (6.70%)	37 (49.30%) **	31 (41.33%) **	<0.001				
CT	19 (25.30%)	30 (40.00%)	28 (37.33%)					
TT	51 (68.00%)	8 (10.70%) **	16 (21.33%) **					
**Genotype**								
CC	5 (6.70%)	37 (49.30%) **	31 (41.30%) **	<0.001	13.63	<0.001	9.86	<0.001
CT + TT	70 (93.30%)	38 (50.70%) **	44 (58.70%) **		(4.95–37.58)		(3.57–27.27)	
**Genotype**								
CT	19 (25.30%)	30 (40.00%)	28 (37.30%)	0.131	1.97	0.055	1.76	0.113
CC + TT	56 (74.70%)	45 (60.00%)	47 (62.70%)		(0.98–3.94)		(0.87–3.54)	
**Genotype**								
TT	51 (68.00%)	8 (10.70%) **	16 (21.30%) **	<0.001	0.06	<0.001	0.128	<0.001
CC + CT	24 (32.00%)	67 (89.30%) **	59 (78.70%) **		(0.02–0.14)		(0.06–0.27)	
**Allele**								
C	29 (19.30%)	104 (69.30%) **	90 (60.00%) **	<0.001	9.43	<0.001	6.26	<0.001
T	121 (80.70%)	46 (30.70%) **	60 (40.00%) **		(5.53–16.08)		(3.72–10.53)	

The data are listed as frequencies (percentages). ** Significant change compared to the control group (*p* < 0.001).

**Table 4:** Genotype and allele frequencies of rs13384548 (G > A) SNP and association with risks of hepatitis C virus (HCV) and hepatocellular carcinoma (HCC) development.

	Control (n = 75)	HCV (n = 75)	HCV-HCC (n = 75)	Odds Ratio (95% CI) of HCV vs. Control	*p*-Value	Odds Ratio (95% CI) of HCC vs. Control	*p*-Value	Odds Ratio (95% CI) of HCC vs. HCV	*p*-Value
**Genotype**									
GG	14 (18.70%)	34 (45.30%)	52 (69.30%)						
GA	32 (42.70%)	23 (30.70%)	16 (21.30%)						
AA	29 (38.70%)	18 (24.00%)	7 (9.30%)						
**Genotype**									
GG	14 (18.70%)	34 (45.30%) **	52 (69.30%) **^, @^	3.61	<0.001	9.85	<0.001	2.73	0.003
GA + AA	61 (81.30%)	41 (54.70%) **	23 (30.70%) **^, @^	(1.73–7.56)		(4.61–21.07)		(1.40–5.32)	
**Genotype**									
GA	32 (42.70%)	23 (30.70%)	16 (21.30%) *	0.59	0.127	0.36	0.005	0.61	0.193
GG + AA	43 (57.30%)	52 (69.30%)	59 (78.70%) *	(0.30–1.16)		(0.18–0.75)		(0.29–1.28)	
**Genotype**									
AA	29 (38.70%)	18 (24.00%)	7 (9.30%) **^, $^	0.50	0.053	0.16	<0.001	0.33	0.016
GG + GA	46 (61.30%)	57 (76.00%)	68 (90.70%) **^, $^	(0.25–1.01)		(0.07–0.40)		(0.13–0.84)	
**Allele**									
G	60 (40.00%)	91 (60.70%) **	120 (80.00%) ^#^	2.31	<0.001	6	<0.001	2.59	<0.001
A	90 (60.00%)	59 (39.30%) **	30 (20.00%) ^#^	(1.46–3.68)		(3.58–10.06)		(1.55–4.35)	

The data are listed as frequencies (percentages). * Significant change compared to the control group (*p* < 0.01). ** Significant change compared to the control group (*p* < 0.001). ^$^ Significant change compared to the HCV group (*p* < 0.05). ^@^ Significant change compared to the HCV group (*p* < 0.01). ^#^ Significant change compared to the control and HCV groups (*p* < 0.001).

## Data Availability

All data generated or analyzed during this study are included in this published article.

## Abbreviations

The following abbreviations are used in this manuscript:

AFP	Alpha-fetoprotein
ALT	Alanine aminotransferase
AST	Aspartate aminotransferase
BCLC	Barcelona Clinic Liver Cancer staging system
CTLA-4	Cytotoxic T-lymphocyte-associated antigen-4
ELISA	Enzyme-linked immunosorbent assay
HCC	Hepatocellular carcinoma
HCV	Hepatitis C virus
MELD/Na	Model for End-Stage Liver Disease Sodium score
OR	Odds ratio
PC	Prothrombin concentration
sCTLA-4	soluble CTLA-4
SNPs	Single-nucleotide polymorphisms
